# Supplementary material for: An architectural role of specific RNA–RNA interactions in oskar granules
Source: Nat Cell Biol. 2024 Oct 1;26(11):1934–42. doi: 10.1038/s41556-024-01519-3 (PMC11567897; doi:10.1038/s41556-024-01519-3)
Supplement: Supplementary file 1 — Reporting Summary [file 41556_2024_1519_MOESM1_ESM.pdf]

nature portfolio

Corresponding author(s): Anne Ephrussi

Last updated by author(s): 15/07/2024

Reporting Summary

Nature Portfolio wishes to improve the reproducibility of the work that we publish. This form provides structure for consistency and transparency in reporting. For further information on Nature Portfolio policies, see our Editorial Policies and the Editorial Policy Checklist.

Statistics

For all statistical analyses, confirm that the following items are present in the figure legend, table legend, main text, or Methods section.

n/a	Confirmed	
☐	☒	The exact sample size (*n*) for each experimental group/condition, given as a discrete number and unit of measurement
☐	☒	A statement on whether measurements were taken from distinct samples or whether the same sample was measured repeatedly
☐	☒	The statistical test(s) used AND whether they are one- or two-sided<br>*Only common tests should be described solely by name; describe more complex techniques in the Methods section.*
☒	☐	A description of all covariates tested
☒	☐	A description of any assumptions or corrections, such as tests of normality and adjustment for multiple comparisons
☐	☒	A full description of the statistical parameters including central tendency (e.g. means) or other basic estimates (e.g. regression coefficient) AND variation (e.g. standard deviation) or associated estimates of uncertainty (e.g. confidence intervals)
☐	☒	For null hypothesis testing, the test statistic (e.g. *F*, *t*, *r*) with confidence intervals, effect sizes, degrees of freedom and *P* value noted<br>*Give P values as exact values whenever suitable.*
☒	☐	For Bayesian analysis, information on the choice of priors and Markov chain Monte Carlo settings
☒	☐	For hierarchical and complex designs, identification of the appropriate level for tests and full reporting of outcomes
☒	☐	Estimates of effect sizes (e.g. Cohen's *d*, Pearson's *r*), indicating how they were calculated

Our web collection on statistics for biologists contains articles on many of the points above.

Software and code

Policy information about availability of computer code

Data collection	Leica SP8 TCS X for image acquisition
Data analysis	Fiji 2.3.0/1.53q, CortAnalyis Fiji plugin, Graph Pad Prism v 9, Huygens Essentials software, Leica Lightning module of Leica SP8 TCS X

For manuscripts utilizing custom algorithms or software that are central to the research but not yet described in published literature, software must be made available to editors and reviewers. We strongly encourage code deposition in a community repository (e.g. GitHub). See the Nature Portfolio guidelines for submitting code & software for further information.

Data

Policy information about availability of data

All manuscripts must include a data availability statement. This statement should provide the following information, where applicable:

- Accession codes, unique identifiers, or web links for publicly available datasets
- A description of any restrictions on data availability
- For clinical datasets or third party data, please ensure that the statement adheres to our policy

Source data are provided with the study in the Supplementary Information file. All other data supporting the findings of this study are available from the corresponding authors on reasonable request.

Research involving human participants, their data, or biological material

Policy information about studies with human participants or human data. See also policy information about sex, gender (identity/presentation), and sexual orientation and race, ethnicity and racism.

Reporting on sex and gender	n/a
Reporting on race, ethnicity, or other socially relevant groupings	n/a
Population characteristics	n/a
Recruitment	n/a
Ethics oversight	n/a

Note that full information on the approval of the study protocol must also be provided in the manuscript.

Field-specific reporting

Please select the one below that is the best fit for your research. If you are not sure, read the appropriate sections before making your selection.

☒ Life sciences ☐ Behavioural & social sciences ☐ Ecological, evolutionary & environmental sciences

For a reference copy of the document with all sections, see nature.com/documents/nr-reporting-summary-flat.pdf

Life sciences study design

All studies must disclose on these points even when the disclosure is negative.

Sample size	For in vivo phenotypic analysis, three biological replicates were analyzed. For in vitro experiments, a minimum of three technical replicates were analyzed. For all experiments sample size was determined according to pilot experiments. Detailed description of sample size is provided in the figure legends.
Data exclusions	No data were excluded from the analysis.
Replication	A minimum of three replicates were performed for each experiment and used for analysis. Experiments provided reproducible data with similar results. Exact number of biological or technical replicates is indicated in the figure legends.
Randomization	Due to the nature of the experiments, no randomization was performed for the data assessment or analysis.
Blinding	Blinding was not performed due to the experimental condition set up. All the comparative samples were analyzed with the same parameters.

Reporting for specific materials, systems and methods

We require information from authors about some types of materials, experimental systems and methods used in many studies. Here, indicate whether each material, system or method listed is relevant to your study. If you are not sure if a list item applies to your research, read the appropriate section before selecting a response.

Materials & experimental systems

n/a	Involved in the study	
☐	☒	Antibodies
☒	☐	Eukaryotic cell lines
☒	☐	Palaeontology and archaeology
☐	☒	Animals and other organisms
☒	☐	Clinical data
☒	☐	Dual use research of concern
☒	☐	Plants

Methods

n/a	Involved in the study	
☒	☐	ChIP-seq
☒	☐	Flow cytometry
☒	☐	MRI-based neuroimaging

Antibodies

Antibodies used	Rabbit anti-Bruno, rabbit anti-Staufen, rabbit anti-PTB, rabbit anti-Egalitarian, mouse anti-Me31B

Validation	Rabbit anti-Bruno: Validated by Western blot of UV-crosslinking and RNA affinity pull down experiments (reference: Besse et al., Drosophila PTB promotes formation of high-order RNP particles and represses oskar translation, Genes & Development 23:195–207 (2009))<br><br>Rabbit anti-Staufen: Validated by immunofluorescence using egg chambers from wild type and staufen alleles (reference: St Johnston, et al., Staufen, a gene required to localize maternal RNAs in Drosophila eggs. Cell 66, 51-63 (1991))<br><br>Rabbit anti-PTB: Validated by Western blot in PTB GFP-trap lines and PTB germline clones (reference: Besse et al., Drosophila PTB promotes formation of high-order RNP particles and represses oskar translation, Genes & Development 23:195–207 (2009))<br><br>Rabbit anti-Egalitarian: Validated by Western blot in Egl mutant alleles (reference: Mach, J. M. & Lehmann, R. An Egalitarian-BicaudalD complex is essential for oocyte specification and axis determination in Drosophila. Genes & development 11, 423-435 (1997))<br><br>Mouse anti-Me31B: Validated by immunofluorescence in Me31B null tissue sample (reference: Nakamura, A., Amikura, R., Hanyu, K. & Kobayashi, S. Me31B silences translation of oocyte-localizing RNAs through the formation of cytoplasmic RNP complex during Drosophila oogenesis. Development 128, 3233–3242 (2001))

Animals and other research organisms

Policy information about studies involving animals; ARRIVE guidelines recommended for reporting animal research, and Sex and Gender in Research

Laboratory animals	Drosophila melanogaster:<br>OregonR, w1118 was used as wild type. OregonR (Bloomington Stock #5) was used for mass extraction of ovaries for RNA affinity capture.<br><br>oskarA87 (Jenny, A. et al. A translation-independent role of oskar RNA in early Drosophila oogenesis. Development 133, 2827-2833, doi:10.1242/dev.02456 (2006)).<br><br>Df(3R)pXT103 (Lehmann, R. & Nusslein-Volhard, C. Abdominal segmentation, pole cell formation, and embryonic polarity require the localized activity of oskar, a maternal gene in Drosophila. Cell 47, 141-152, doi:10.1016/0092- 8674(86)90375-2 (1986)).<br><br>oskarattP,3P3-GFP (Gaspar, I. et al., An RNA-binding atypical tropomyosin recruits kinesin-1 dynamically to oskar mRNPs. EMBO J 36, 319-333, doi:10.15252/embj.201696038 (2017)).<br><br>EGFP:Me31B (Nakamura, A. et al., Me31B silences translation of oocyte-localizing RNAs through the formation of cytoplasmic RNP complex during Drosophila oogenesis. Development, 128, 3233–3242 (2001)).<br><br>oskar WT (Hachet, O. & Ephrussi, A. Splicing of oskar RNA in the nucleus is coupled to its cytoplasmic localization. Nature 428, 959-963, (2004)).<br><br>oskar UU (Jambor, H. et al., Dimerization of oskar 3' UTRs promotes hitchhiking for RNA localization in the Drosophila oocyte. RNA 17, 2049-2057, (2011)).<br><br>oskarGAL4 (Bloomington Stock #44242).<br><br>BrunoKI-EGFP (DGRC # 118625).
Wild animals	No wild animals were used in this study.
Reporting on sex	Reporting on sex was not applicable in this study.
Field-collected samples	No samples were collected from the field.
Ethics oversight	No ethical approval was needed for this study.

Note that full information on the approval of the study protocol must also be provided in the manuscript.
