# Supplementary figures and images for: An architectural role of specific RNA–RNA interactions in oskar granules
Source: Nat Cell Biol. 2024 Oct 1;26(11):1934–42. doi: 10.1038/s41556-024-01519-3 (PMC11567897; doi:10.1038/s41556-024-01519-3)

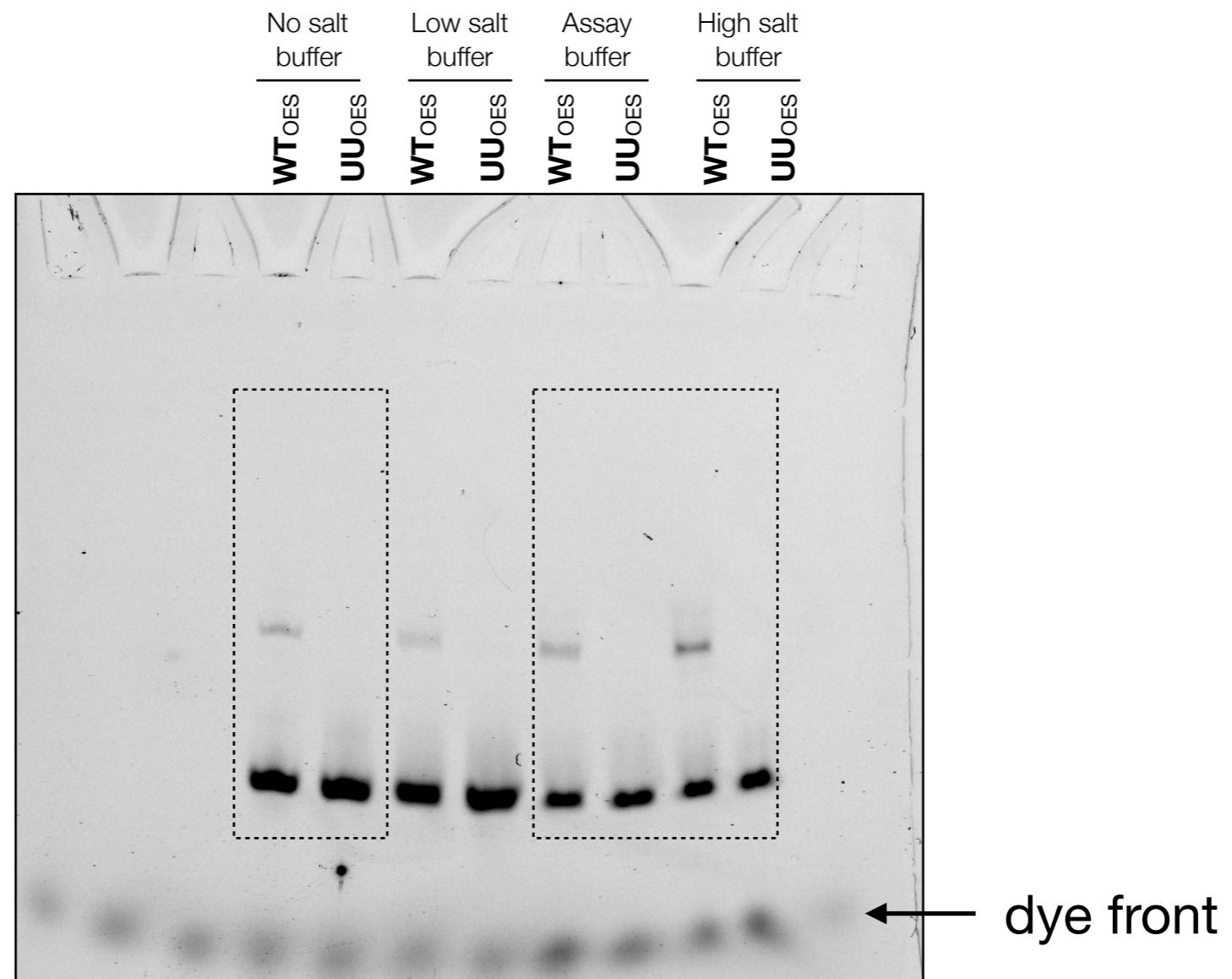

Fig. 1b

Supplement: Supplementary file 4 — Unprocessed gels. [file 41556_2024_1519_MOESM4_ESM.pdf]

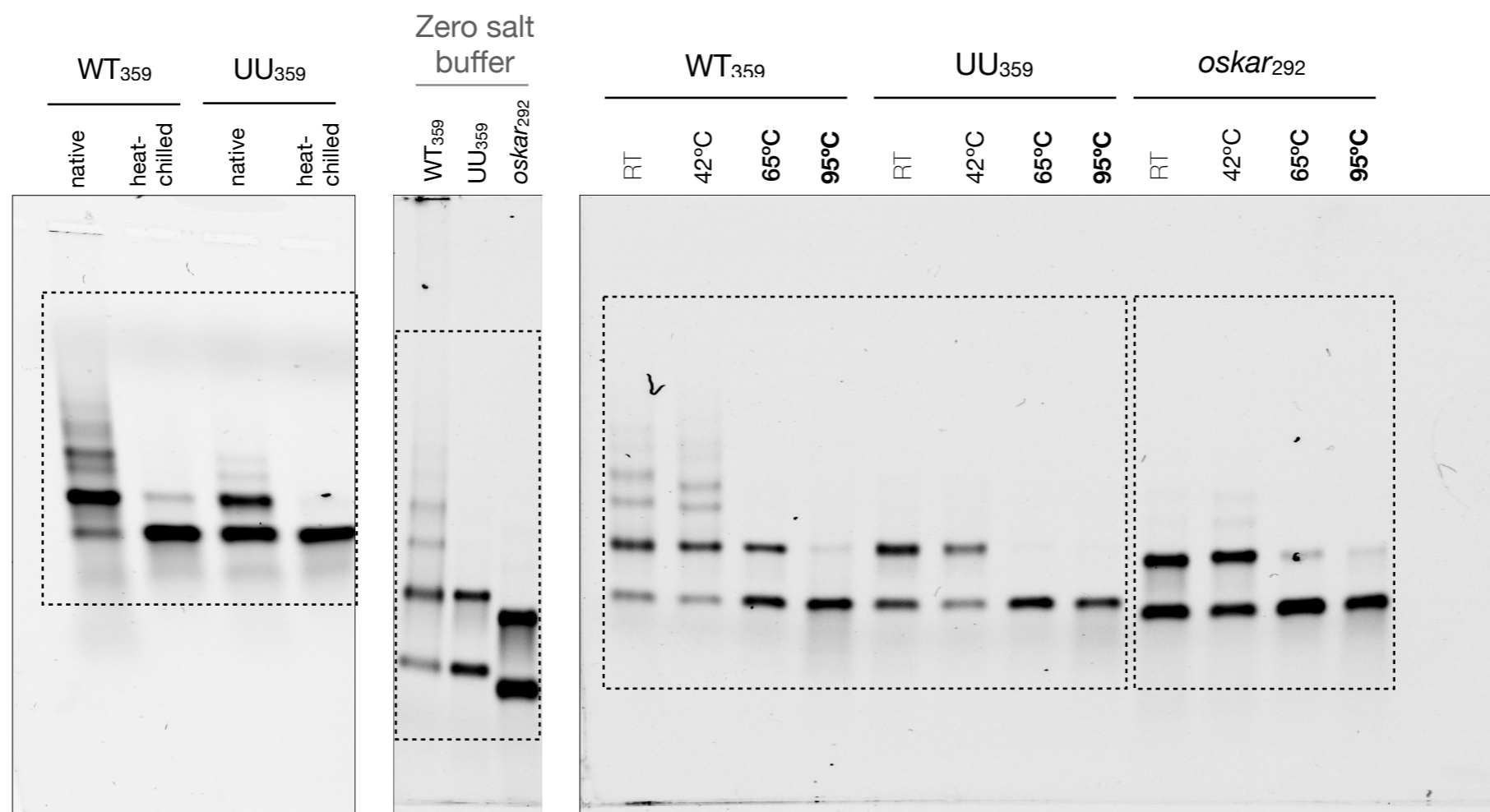

Fig. 2a

Fig. 2c

Supplement: Supplementary file 6 — Unprocessed gels. [file 41556_2024_1519_MOESM6_ESM.pdf]

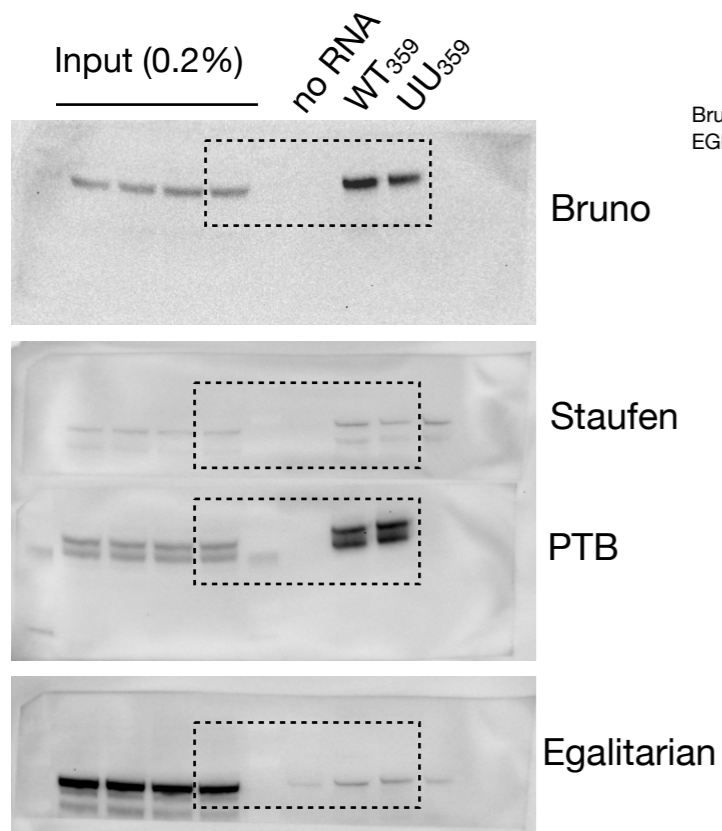

Fig. 3a

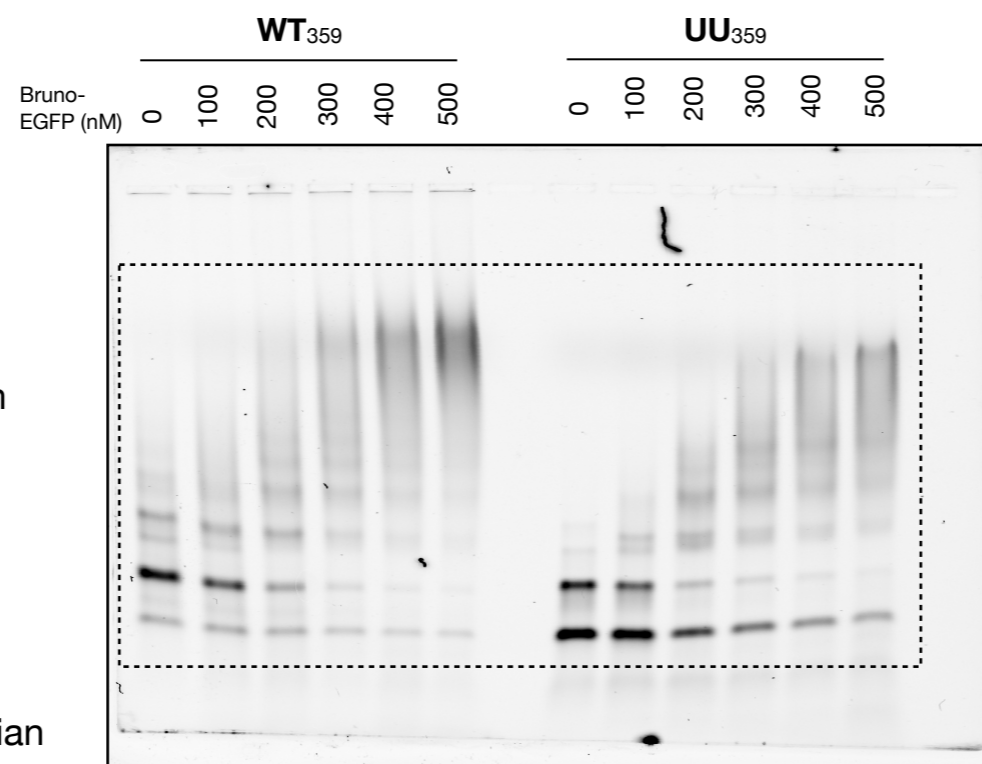

Fig. 3c

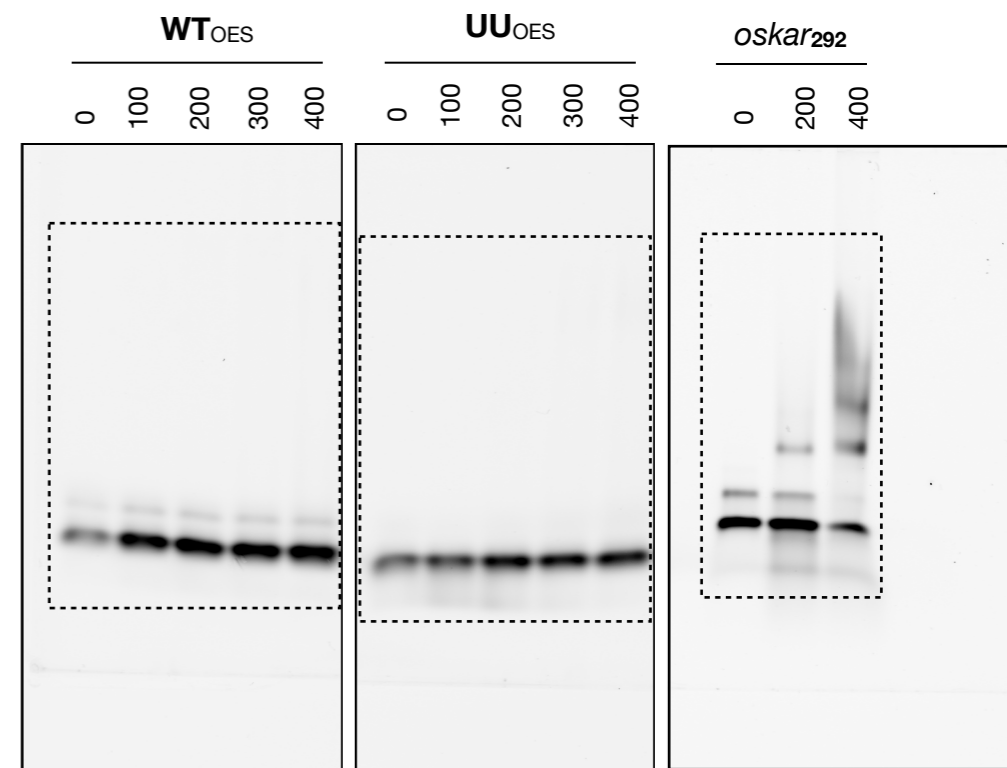

Fig. 3d

Supplement: Supplementary file 8 — Unprocessed gels. [file 41556_2024_1519_MOESM8_ESM.pdf]

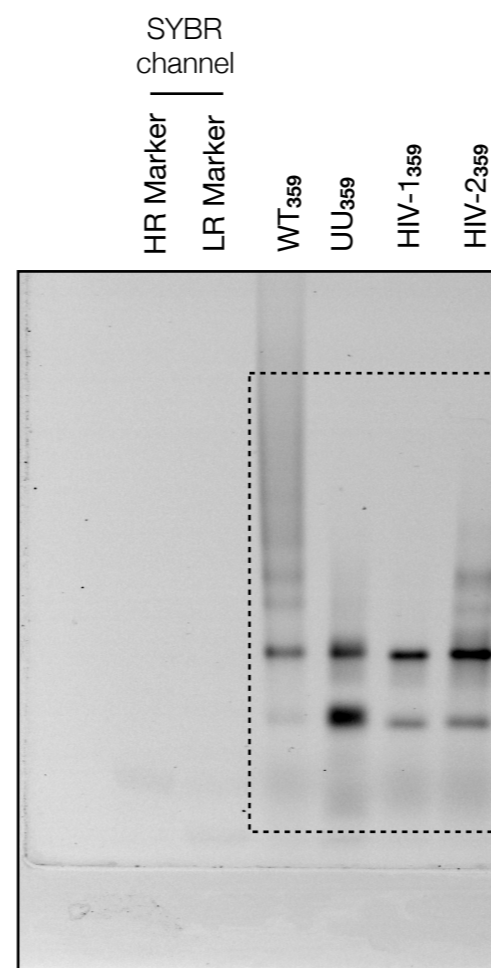

Fig. 4d

Supplement: Supplementary file 10 — Unprocessed gels. [file 41556_2024_1519_MOESM10_ESM.pdf]

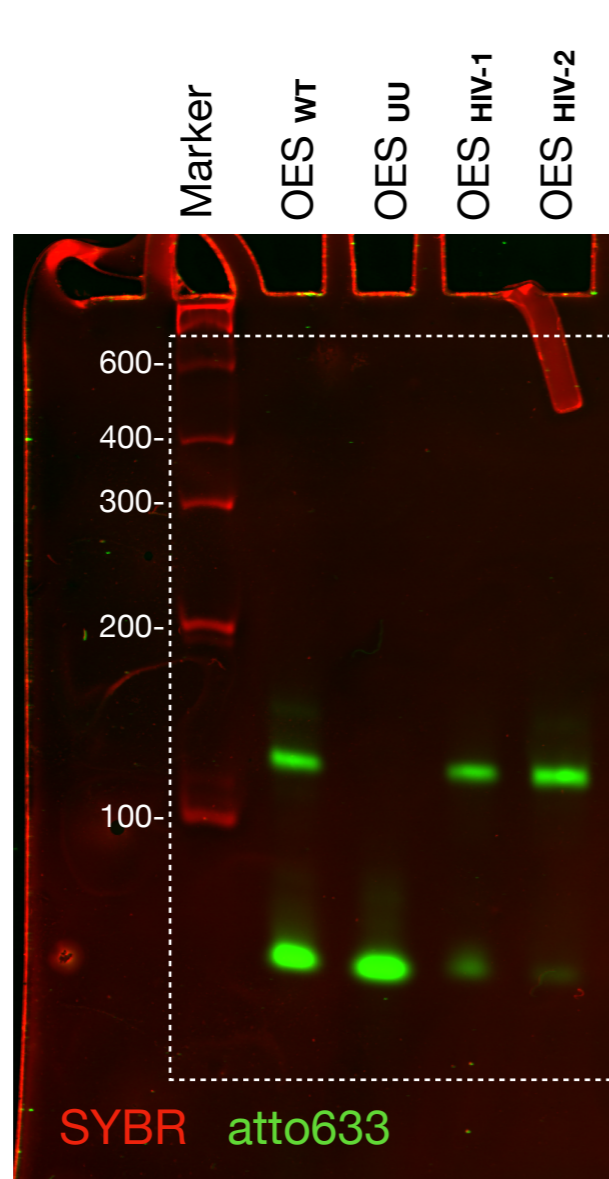

Extended Data Fig. 1a

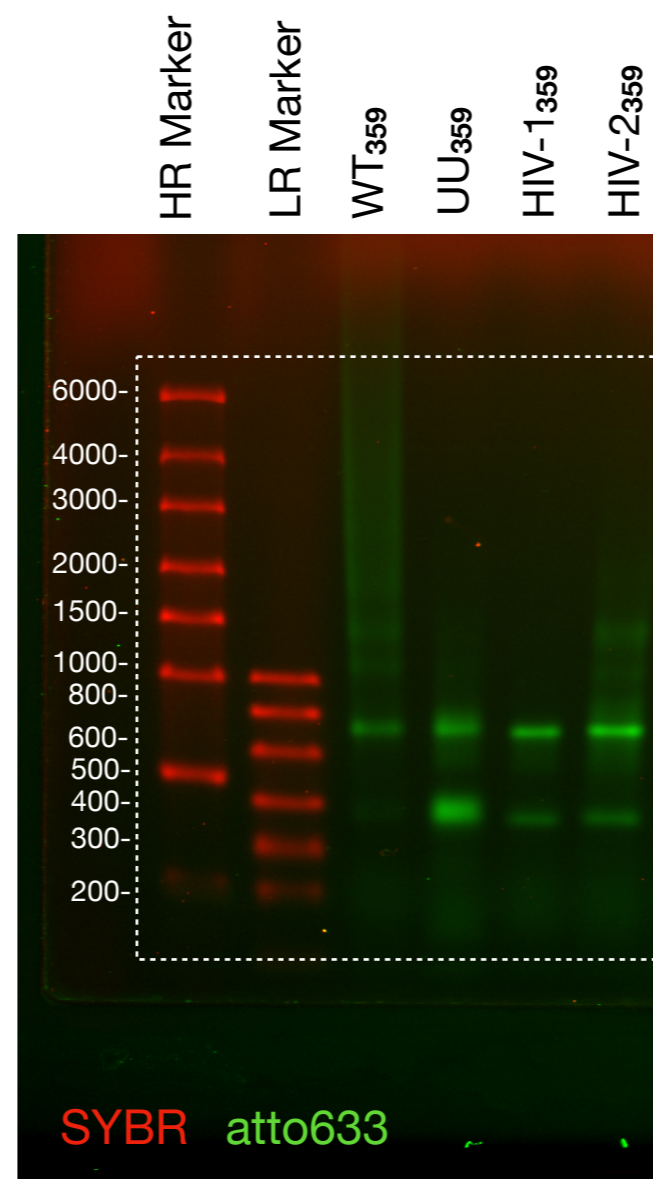

Extended Data Fig. 1b

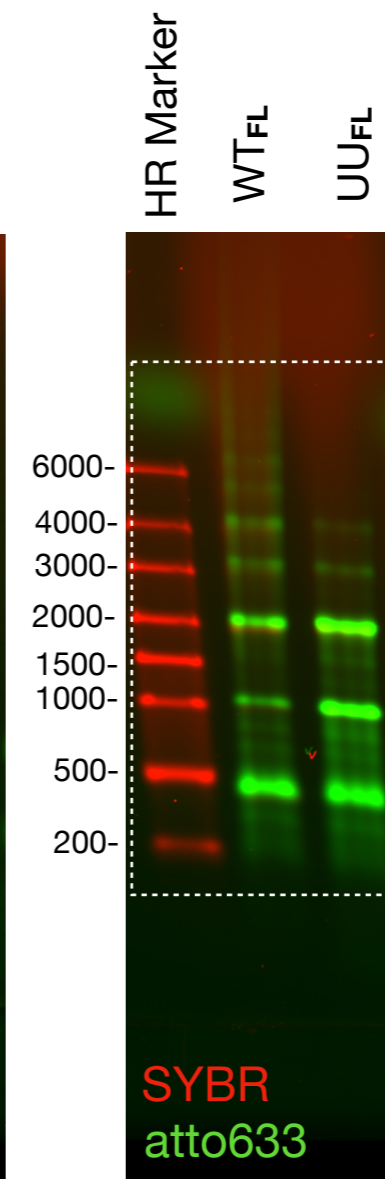

Extended Data Fig. 1c

Supplement: Supplementary file 13 — Unprocessed gels. [file 41556_2024_1519_MOESM13_ESM.pdf]

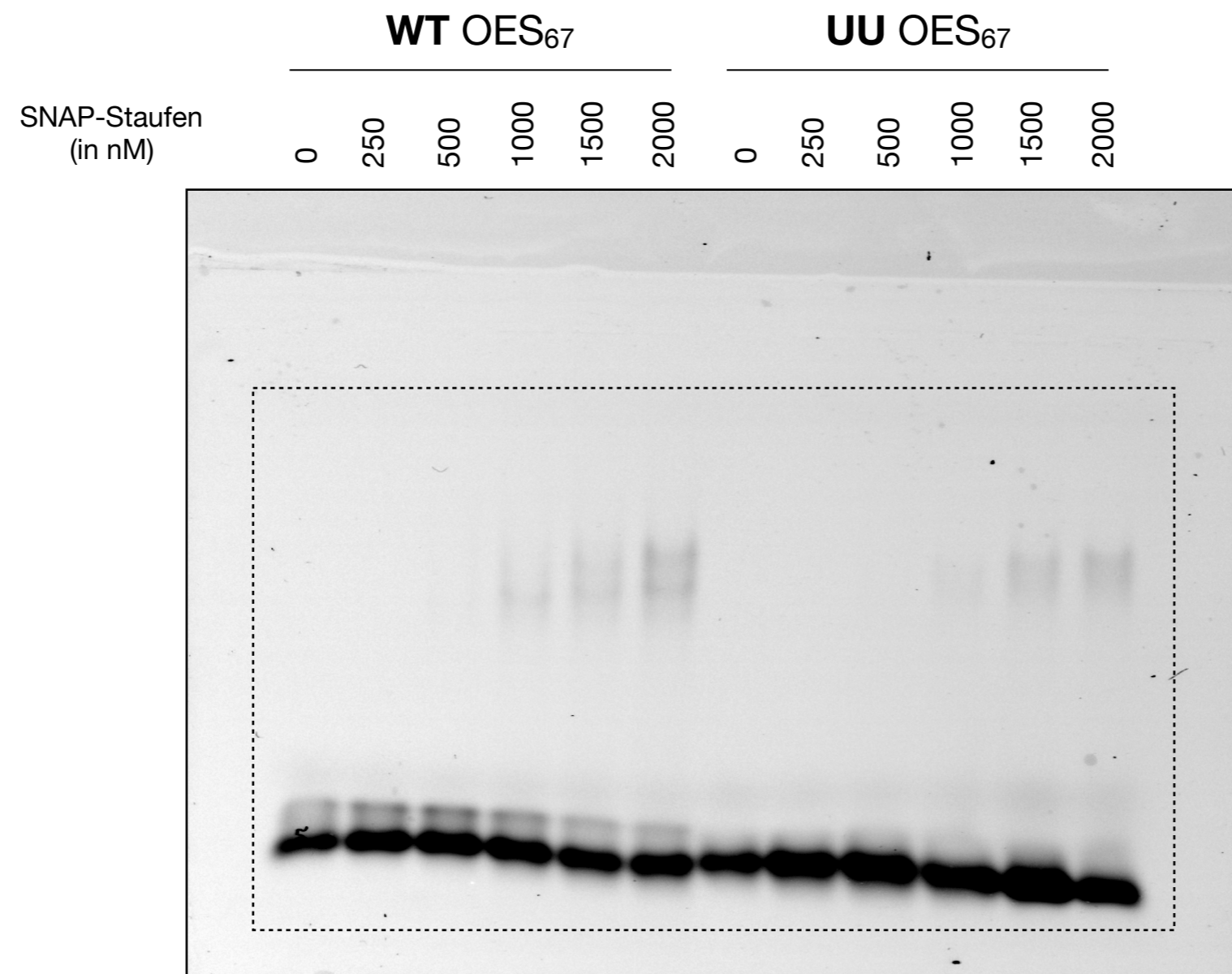

Extended Data Fig. 4b

Supplement: Supplementary file 15 — Unprocessed gels. [file 41556_2024_1519_MOESM15_ESM.pdf]

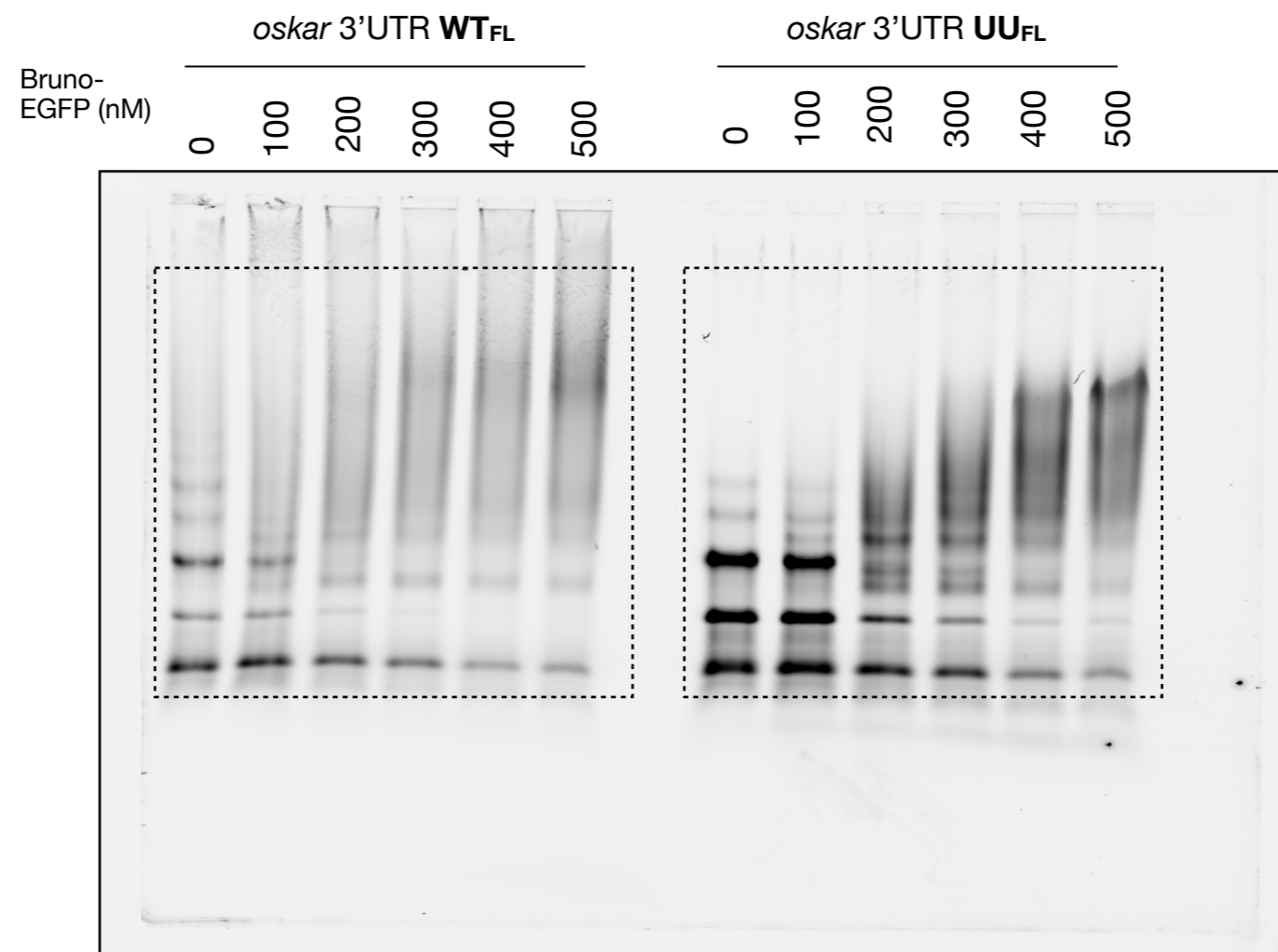

Extended Data Fig. S5

Supplement: Supplementary file 17 — Unprocessed gels. [file 41556_2024_1519_MOESM17_ESM.pdf]

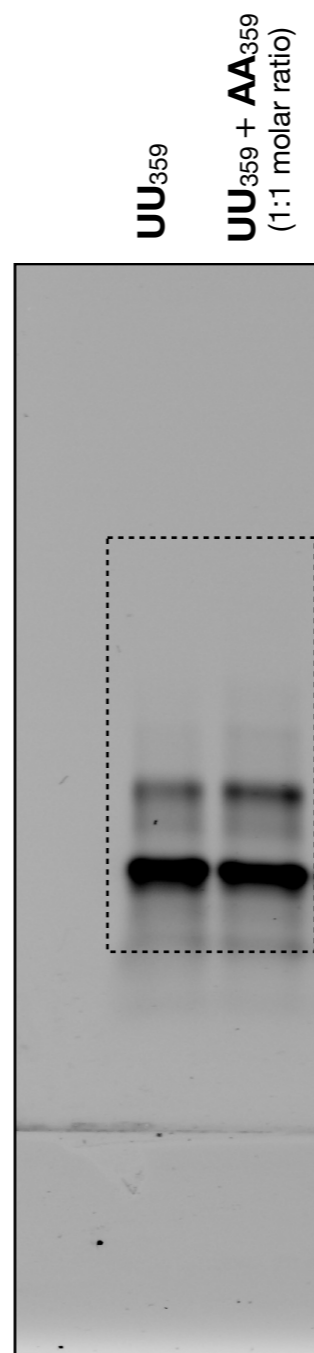

Extended Data Fig. 6b

Supplement: Supplementary file 19 — Unprocessed gels. [file 41556_2024_1519_MOESM19_ESM.pdf]
